# Sample size determination for mediation analysis of longitudinal data

**DOI:** 10.1186/s12874-018-0473-2

**Published:** 2018-03-27

**Authors:** Haitao Pan, Suyu Liu, Danmin Miao, Ying Yuan

**Affiliations:** 1 Department of Biostatistics, St. Jude Children’s Research Hospital, Memphis, TN 38105 USA; 2 Department of Biostatistics, Department of Biostatistics, Unit 1411, Anderson Cancer Center, The University of Texas MD, 1515 Holcombe Blvd, Houston, TX 77030 USA; 3 Department of Medical Psychology, Fourth Military Medical University, Xi’an, 710032 China

**Keywords:** Sample size determination, Mediation analysis, Longitudinal study

## Abstract

**Background:**

Sample size planning for longitudinal data is crucial when designing mediation studies because sufficient statistical power is not only required in grant applications and peer-reviewed publications, but is essential to reliable research results. However, sample size determination is not straightforward for mediation analysis of longitudinal design.

**Methods:**

To facilitate planning the sample size for longitudinal mediation studies with a multilevel mediation model, this article provides the sample size required to achieve 80% power by simulations under various sizes of the mediation effect, within-subject correlations and numbers of repeated measures. The sample size calculation is based on three commonly used mediation tests: Sobel’s method, distribution of product method and the bootstrap method.

**Results:**

Among the three methods of testing the mediation effects, Sobel’s method required the largest sample size to achieve 80% power. Bootstrapping and the distribution of the product method performed similarly and were more powerful than Sobel’s method, as reflected by the relatively smaller sample sizes. For all three methods, the sample size required to achieve 80% power depended on the value of the ICC (i.e., within-subject correlation). A larger value of ICC typically required a larger sample size to achieve 80% power. Simulation results also illustrated the advantage of the longitudinal study design. The sample size tables for most encountered scenarios in practice have also been published for convenient use.

**Conclusions:**

Extensive simulations study showed that the distribution of the product method and bootstrapping method have superior performance to the Sobel’s method, but the product method was recommended to use in practice in terms of less computation time load compared to the bootstrapping method. A R package has been developed for the product method of sample size determination in mediation longitudinal study design.

**Electronic supplementary material:**

The online version of this article (10.1186/s12874-018-0473-2) contains supplementary material, which is available to authorized users.

## Background

**Fig. 1:**
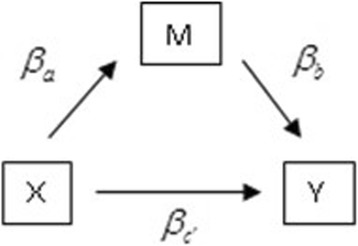
Path diagram for simple single-level mediation model

Mediation analysis is a statistical method that helps researchers to understand the mechanisms underlying the phenomena they study. It has broad application in psychology, prevention research, and other social sciences. A simple mediation framework (see Fig. 1) involves three variables: the independent variable, dependent variable and mediating variable [4, 27]. The aim of mediation analysis is to determine whether the relation between the independent and dependent variables is due, wholly or in part, to the mediating variables. Since the seminal work of Baron and Kenney [4], extensive research has been conducted in mediation analysis, including that of [7, 22, 25]; [34]; and [18], among others. A comprehensive review of mediation analysis can be found in the book by [27].

When planning a mediation study, the investigator commonly determines the required sample size. An appropriately chosen sample size is critical for the success of the study. If the sample size is too small, the study may lack adequate statistical power to detect an effect size of practical importance, which leads the investigator to incorrectly conclude that an efficacious intervention is inefficacious. Reviews of the psychological literature suggest that insufficient statistical power is a common problem in psychological studies [1, 29, 30]. On the other hand, an unnecessarily large sample size is wasteful and increases the duration of the study. Because of the importance of sample size, funding agencies such as the National Institutes of Health routinely require investigators to justify the sample size for funded projects.

Unfortunately, sample size determination is not straightforward for mediation analysis. No simple formula is available to carry out this task. Using Monte Carlo simulations, Fritz and MacKinnon [14] investigated power calculations for the simple mediation model and provided guidance in choosing sample sizes for mediation studies with independent data. Their results, however, are not applicable to longitudinal studies, in which data are correlated.

A longitudinal study design is common in psychological and social research [13]. Compared with a cross-sectional study design, the longitudinal design requires fewer subjects and allows investigators to study the trajectory of each subject. In longitudinal studies, repeated measures are collected from each subject over time. Since measures collected from the same subject are more likely to be similar when compared to those collected from other subjects, data from the same subject tend to be correlated. Analyzing such correlated data requires special statistical methods, such as the multilevel model [33]. In this article, assuming a multilevel mediation model and using Monte Carlo simulation, we investigate sample size determination for longitudinal mediation studies. Our objective is to provide practical guidance and easy-to-use R software to help researchers determine the sample size when designing longitudinal mediation studies.

## Methods

This section starts by formulating single-level mediation model, then multilevel mediation model for longitudinal data is described. We focus on lower-level multilevel mediation model and relevant model assumptions are discussed.

### Simple single-level mediation model

Let *Y* denote the dependent (or outcome) variable, *X* denote the independent variable, and *M* denote the mediating variable (or mediator). A single-level mediation model (Fig. 1) can be expressed in the form of three regression equations:

1 $$ Y={\beta}_{01}+{\beta}_cX+{\varepsilon}_1 $$

2 $$ Y={\beta}_{02}+{\beta}_{c^{\prime }}X+{\beta}_bM+{\varepsilon}_2 $$

3 $$ M={\beta}_{03}+{\beta}_aX+{\varepsilon}_3, $$

where *β*_*c*_ quantifies the relation between the independent variable and dependent variable (i.e., the total effect of *X* on *Y)*; ${\beta}_{c^{\prime }}$ quantifies the relation between the independent variable and dependent variable after adjusting for the effect of the mediating variable (i.e., the direct effect of *X* on *Y* adjusted for *M)*; *β*_*b*_ quantifies the relation between the mediating variable and dependent variable after adjusting for the effects of the independent variable; *β*_*a*_ measures the relation between the independent variable and mediating variable; *β*_01_, *β*_02_, and *β*_03_ are intercepts; and *ε*_1_, *ε*_2_, and *ε*_3_ are error terms that follow normal distributions with mean 0 and respective variances of ${\sigma}_1^2,{\sigma}_2^2$, and ${\sigma}_3^2$.

The mediation effect can be defined by two ways: *β*_*c*_ − *β*_*c*'_ and *β*_*a*_*β*_*b*_ [16, 17, 27]. For the single-level mediation model, the two definitions of the mediation effect are equivalent [28], but they are generally different in the multilevel mediation models we will describe.

### Multilevel mediation model for longitudinal data

For correlated longitudinal data, the simple mediation model, which assumes independence of observations, is not appropriate. Using the single-level mediation model for longitudinal data leads to biased estimates of standard errors and confidence intervals [3].

Multilevel mediation modeling is a powerful technique for analyzing mediation effects in longitudinal data. Multilevel models assume that there are at least two levels in the data, an upper level and a lower level. The lower-level units (e.g., repeated measures) are often nested within the upper-level units (e.g., subjects). Assuming that the lower-level units are random, also known as random effects, multilevel models appropriately account for correlations among the observations from the same subject, and yield valid statistical inference. For a comprehensive coverage of multilevel modeling techniques, see the book by Raudenbush & Bryk [33].

**Fig. 2:**
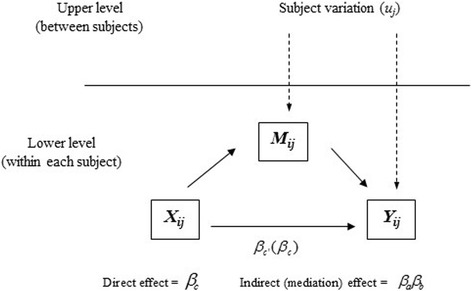
Pathway diagram for a 1–1-1 mediation model

The multilevel mediation model is much more complex than the single-level model because mediation effects can occur at the different model levels. Two kinds of mediation, upper-level mediation and lower-level mediation, can be distinguished in the context of multilevel mediation models [5]. In upper-level mediation, the initial causal variable for which the effect is mediated is an upper-level variable. In lower-level mediation, the mediator is a lower-level variable. Krull [21] and MacKinnon [22] offered examples of upper-level mediation, while [18] studied lower-level mediation, in which the mediation links varied randomly across the upper-level units. In this study, we focus on a specific type of lower-level mediation model (Fig. 2) that is appropriate for analyzing longitudinal studies. In this model, an initial variable X is mediated in the lower level (i.e., measurement level), but the mediator M and outcome Y are affected by upper-level (i.e., subject level) variations. A simple scenario for this model is a longitudinal experimental study in which subjects are randomly assigned to a treatment (time-invariant) or the multiple treatments can be assigned to a same subject in cross-over design (i.e., initial variable X, in this paper, variable X is treated as time-varying), and mediating variable M, such as a psychosocial measure, is believed to change individual behavior (i.e., dependent variable Y) over time.

### The lower-level mediation model

Let *X*_*ij*_, *Y*_*ij*_, and *M*_*ij*_ denote the independent variable, dependent variable, and mediating variable, respectively, for the *i*th observation from the *j*th subject. The lower-level mediation model in Fig. 2 can be expressed in the form of the following two-level regression equations,

4 $$ Lower:{Y}_{ij}={\beta}_{01j}+{\beta}_c{X}_{ij}+{\varepsilon}_{1 ij} $$

5 $$ Upper:{\beta}_{01j}={\gamma}_1+{u}_{1j} $$

6 $$ Lower\kern0.5em :{Y}_{ij}={\beta}_{02j}+{\beta}_{c\hbox{'}}{X}_{ij}+{\beta}_b{M}_{ij}+{\varepsilon}_{2 ij} $$

7 $$ Upper:{\beta}_{02j}={\gamma}_2+{u}_{2j} $$

8 $$ Lower:{M}_{ij}={\beta}_{03j}+{\beta}_a{X}_{ij}+{\varepsilon}_{3 ij} $$

9 $$ Upper:{\beta}_{03j}={\gamma}_3+{u}_{3j} $$

where at the lower (or within-subject) level, similar to the simple single-level mediation model, *β*_*c*_ measures the total effect of the independent variable on the dependent variable; *β*_*c*'_ measures the direct effect of the independent variable on the dependent variable, adjusted for the mediating variable; *β*_*b*_ measures the effect of the mediating variable on the dependent variable, adjusted for the independent variable; *β*_*a*_ measures the effect of the independent variable on the mediating variable; and *β*_01*j*_, *β*_02*j*_, and *β*_03*j*_ are subject-specific intercepts that differ from subject to subject, as reflected by the subscript *j* in these parameters. These subject-specific intercepts are also known as random intercepts. The terms *ε*_1*ij*_, *ε*_2*ij*_, and *ε*_3*ij*_ are lower-level (or within-subject) error terms that follow normal distributions with a mean of zero and respective variances ${\sigma}_1^2,{\sigma}_2^2$, and ${\sigma}_3^2$. At the upper (or between-subject) level *γ*_1_, *γ*_2_, and *γ*_3_ are overall or population average intercepts; and *u*_1*j*_, *u*_2*j*_, and *u*_3*j*_ are upper-level (between-subject) error terms that follow normal distributions with a mean of zero and respective variances ${\tau}_1^2,{\tau}_2^2$, and ${\tau}_3^2$.

In the multilevel model, the upper-level errors induce within-subject correlations. Let *y*_*ij*_ and ${y}_{i^{\prime_j}}$ denote the *i-th* and *i*^′^-th measures for the same subject *j*, then *y*_*ij*_ and ${y}_{i^{\prime_j}}$ are correlated as

$$ {\displaystyle \begin{array}{l}\operatorname{cov}\left({y}_{ij},{y}_{i^{\prime_j}}\right)=\operatorname{cov}\left({\beta}_{02j}+{\beta}_{c\hbox{'}}{X}_{ij}+{\beta}_b{M}_{ij}+{\varepsilon}_{2 ij},{\beta}_{02j}+{\beta}_{c\hbox{'}}{X}_{i\hbox{'}j}+{\beta}_b{M}_{i\hbox{'}j}+{\varepsilon}_{2{i}^{\prime }j}\right)\\ {}\kern4.5em =\operatorname{cov}\left({\beta}_{02j},{\beta}_{02j}\right)={\tau}_2^2\end{array}} $$

Such within-subject correlation is often measured by the intraclass correlation coefficient (ICC), which is defined as

$$ ICC=\frac{\mathrm{within}\hbox{-} \mathrm{subject}\ \mathrm{covariance}}{\mathrm{overall}\ \mathrm{variance}} $$

Under the above two-level mediation model, the value of ICC for *Y* is given by

10 $$ ICC={\frac{\tau_2^2}{\sigma_2^2+{\tau}_2^2}}_{.} $$

Larger values of ICC represent strong within-subject correlations, i.e., measures from the same subject are more similar. When ICC = 0, measures from the same subject are independent.

Due to the within-subject correlation, the two definitions of the mediation effects, *β*_*c*_ − *β*_*c*'_ and *β*_*a*_*β*_*b*_, are generally not equivalent in multilevel models [21], although they are equivalent in the single-level mediation model. The different behaviors of multilevel and single-level models are caused by the fact that the weighting matrix used to estimate the multilevel model is typically not identical to single-level equations. The non-equivalence between *β*_*c*_ − *β*_*c*'_ and *β*_*a*_*β*_*b*_, however, is unlikely to be problematic because the difference between the two estimates is typically small and unsystematic and tends to vanish at large sample sizes [21]. In this article, we focus on *β*_*a*_*β*_*b*_ as the measure of the mediation effect.

## Test of the mediation effect

As the independence assumption is violated, conventional statistical methods, such as the ordinary least squares method, are not appropriate for estimating the multilevel mediation model. Instead, maximum likelihood methods and/or empirical Bayes methods are typically used. Let ${\widehat{\beta}}_a$ and ${\widehat{\beta}}_b$ denote the maximum likelihood estimates of *β*_*a*_ and *β*_*b*,_ respectively. Then, the maximum likelihood estimate of the mediation effect is given by ${\widehat{\beta}}_a{\widehat{\beta}}_b$. To test whether the mediation effect *β*_*a*_*β*_*b*_ equals zero, three approaches can be taken.

### Sobel’s method

Sobel’s method is a widely used test of the mediation effect, based on the first-order multivariate delta method [35, 36]. In this approach, assuming ${\widehat{\beta}}_a$and ${\widehat{\beta}}_b$are independent, the standard deviation of ${\widehat{\beta}}_a{\widehat{\beta}}_b$ is estimated by

11 $$ {\widehat{s}}_{\beta_a{\beta}_b}=\sqrt{{\widehat{s}}_{\beta_a}^2{\widehat{\beta}}_b^2+{\widehat{s}}_{\beta_b}^2{\widehat{\beta}}_a^2}, $$

where ${\widehat{s}}_{\beta_a}^2$and ${\widehat{s}}_{\beta_b}^2$are the squared standard errors of ${\widehat{\beta}}_a$ and ${\widehat{\beta}}_b$, respectively. The 100(1-*α*)% confidence interval (CI) of the mediation effect is given by

12 $$ \left({\widehat{\beta}}_a{\widehat{\beta}}_b-{z}_{1-\alpha /2}{\widehat{s}}_{\beta_a{\beta}_b},{\widehat{\beta}}_a{\widehat{\beta}}_b+{z}_{1-\alpha /2}{\widehat{s}}_{\beta_a{\beta}_b}\right), $$

where *z*_1 − *α*/2_ is the (1 − *α*/2)th quantile of the standard normal distribution. If *α* = 0.05, the familiar 95% CI results. If this CI does not contain zero, we reject the null hypothesis and conclude that the mediation effect is statistically significant.

Sobel’s method relies on the assumption that ${\widehat{\beta}}_a{\widehat{\beta}}_b$, the product of two normal random variables ${\widehat{\beta}}_a$ and ${\widehat{\beta}}_b$, is normally distributed. However, several studies have shown that the distribution of the product of two normal random variables is not actually normal, but skewed [23]. The violation of the normality assumption compromises the performance of Sobel’s method and leads to invalid CIs [26]. To address this problem, [26] discussed several improved CIs that account for the fact that ${\widehat{\beta}}_a{\widehat{\beta}}_b$ is not normally distributed, including the CI based on the distribution of the product of two normal random variables and the CI based on the bootstrap method [6, 34].

### Distribution of the product method

Instead of assuming the normality of ${\widehat{\beta}}_a{\widehat{\beta}}_b$, the distribution of the product method proposed by MacKinnon and Lockwood (2001) constructs the CI of the mediation effect based on the distribution of the product of two normal random variables. Although such a distribution does not take a simple closed form, Meeker et al. [31] provided tables of critical values for this distribution that can be used to construct the CI. Alternatively, the critical values can also be obtained based on the empirical distribution of the product of two normal random variables through Monte Carlo simulations. Let *δ*_*lower*_ and *δ*_*upper*_ denote critical values that correspond to the lower and upper bounds of the CI, then the CI of the mediation effect is given by

13 $$ \left({\widehat{\beta}}_a{\widehat{\beta}}_b-{\delta}_{lower}\times {\widehat{s}}_{\beta_a{\beta}_b},{\widehat{\beta}}_a{\widehat{\beta}}_b+{\delta}_{upper}\times {\widehat{s}}_{\beta_a{\beta}_b}\right). $$

### Bootstrap method

Another approach for constructing the CI without imposing a normal assumption on ${\widehat{\beta}}_a{\widehat{\beta}}_b$ is the bootstrap method [11]. The bootstrap method, based on resampling, is useful for finding the standard error and forming CIs for estimates when their sampling distributions are unknown. In this study, we use the percentile bootstrap [6] to construct the CI for the mediation effect. We repeatedly resample the original data with replacement, obtaining the so-called bootstrap samples. For each of the bootstrap samples, we estimate the mediation effect using the maximum likelihood method. These estimates form the empirical distribution of the mediation effect. Let *q*_*α*/2_ and *q*_1 − *α*/2_ denote the (*α*/2)th and (1 − *α*/2)th percentiles of this empirical distribution; then the 100(1 − *α*)% CI of the mediation effect is given by

14 $$ \left({q}_{\alpha /2},{q}_{1-\alpha /2}\right). $$

When conducting bootstrap resampling for the multilevel mediation model, in principle, we should resample both the upper-level (subjects) and lower-level (measures) units. However, in a multilevel context, we should be careful of not breaking the structure of the dataset, therefore, a resampling scheme for multilevel models must take into account the hierarchical data structure. There are three approaches can be applied to bootstrap two-level models: the parametric bootstrap, the residual bootstrap, and the cases bootstrap. We chose the cases bootstrap since it requires minimal assumptions of hierarchical dependency in the data being assumed to be specified correctly. de Leeuw & Meijer [9] suggest that when the number of lower-level units (measures) is small, the approach of resampling only the upper level and keeping the lower level intact yields more accurate estimates. In our simulation, the number of lower-level units is small (i.e., 2 to 5), thus we only resampled the upper-level units. To be specific, the algorithm for cases bootstrap is as follows:

1. Draw a sample of size J with replacement from the upper level units; that is, draw a sample {${j}_k^{\ast },k=1,\cdots, J$} (with replacement) of upper level numbers.
2. For each k, draw a sample of entire cases, with replacement, from (the original) upper level unit $j={j}_k^{\ast }$. This sample has the same size ${n}_k^{\ast }={n}_{j_k^{\ast }}={n}_j$ as the original unit from which the cases are drawn. Then, for each k, we have a set of data {(${Y}_{ik}^{\ast },{X}_{ik}^{\ast },{M}_{ik}^{\ast }$),$i=1,\cdots, {n}_k^{\ast }$}.
3. Compute estimates for all parameters of the two-level model.
4. Repeat steps 1–3 B times.

## Simulation study

We conducted a simulation study to determine the sample size that is needed to achieve 80% power when using Sobel’s method, the distribution of the product method, and the bootstrap method for longitudinal mediation studies. In our simulation, we varied three factors. The first one is the effect size of the mediation effect ${\widehat{\beta}}_a{\widehat{\beta}}_b$. We considered four values of *β*_*a*_ and *β*_*b*_: 0.14, 0.26, 0.39 and 0.59, respectively corresponding to smaller, medium, halfway (between medium and large), and large effect sizes. These values yielded 16 combinations of effect sizes of the mediation effect. Another factor is the ICC. We considered five values of ICC, 0.1, 0.3, 0.5, 0.7 and 0.9, to cover various within-subject correlations from low to high. The last factor is the number of repeated measures. We considered 2, 3, 4 and 5 repeated measures for each subject. For other parameters, we set the overall intercepts*γ*_2_and *γ*_3_ as zero. Since there were no repeated measurements in Fritz et al. [14] and the samples were all drawn from a standard normal distribution, for fair comparisons, we set marginal variances of *Y*_*ij*_ and *M*_*ij*_, that is, ${\sigma}_2^2+{\tau}_2^2$ and ${\sigma}_3^2+{\tau}_3^2$, as 1. Based on the definition of ICC, we have${\tau}_2^2={\tau}_3^2= ICC$.

To simulate data, we first simulated the independent variable *X* from the standard normal distribution, then generated random intercepts *β*_02*j*_ and *β*_03*j*_ according to eqs. (7) and (9). Conditional on the values of *β*_02*j*_ and *β*_03*j*_, we generated the dependent variable *Y* and mediating variable *M* according to eqs. (6) and (8).

To determine the power of the three test methods, under each of the parameter settings, we generated 1000 simulated datasets, and applied the methods to each of the datasets to test the mediation effect. We calculated the power of the methods as the proportion of tests that rejected the null hypothesis of no mediation effects, i.e., the CI excluded zero. For the bootstrap method, we based the construction of the CI on 500 bootstrap samples.

To determine the sample size that yields 80% power, we started with an initial guess of the sample size. If we found the power achieved with that sample size to be too low, we increased the sample size; and if we found the power to be too high, we decreased the sample size. We repeated this procedure until the sample size allowed us to reach the level of power nearest to 80%.

## Results

Tables 1, 2, 3, 4 and 5 show the sample sizes necessary to achieve 80% power under five different ICCs (ICC = 0.1, 0.2, 0.4, 0.6, 0.9). For completeness, results with other ICCs, say, 0.3, 0.5, 0.7, and 0.8, are also shown, which can be found in the Additional file 1: Tables S1- S4, respectively. In each table, the 16 mediation effect sizes are denoted by two letters, with the first one referring to the size of *β*_*a*_, and the second letter referring to the size of *β*_*b*_. We use S for small (0.14), M for medium (0.39), L for large (0.59) and H for halfway (0.26) between large and medium effect sizes, e.g., the effect size ML indicates *β*_*a*_= 0.39 and *β*_*b*_= 0.59.

**Table 1:** Estimated numbers of required subjects for 2, 3, 4 and 5 observations with ICC = 0.1

Observations												
	2			3			4			5		
Effect size^a^	Sobel	Product	Bootstrap	Sobel	Product	Bootstrap	Sobel	Product	Bootstrap	Sobel	Product	Bootstrap
SS	365	299	304	257	215	215	207	163	169	169	136	142
SH	272	235	237	194	172	180	151	140	150	133	126	131
SM	248	226	230	188	188	200	146	144	145	132	126	130
SL	238	248	251	176	176	176	148	147	149	124	126	128
HS	238	201	209	161	138	143	123	104	108	99	83	85
HH	109	88	94	78	65	69	61	50	57	51	42	40
HM	87	77	99	58	55	57	49	42	52	42	40	40
HL	74	72	73	57	51	25	47	46	20	40	38	39
MS	215	200	209	138	134	140	110	102	105	86	83	85
MH	79	65	69	54	46	46	42	35	36	35	29	31
MM	51	41	44	38	30	33	29	23	28	24	21	23
ML	40	36	38	29	27	28	24	22	25	20	18	20
LS	204	206	204	139	132	140	105	101	103	83	82	82
LH	65	60	69	44	41	40	34	31	32	28	24	24
LM	36	31	33	25	22	24	19	17	18	17	15	15
LL	24	20	22	17	16	15	14	13	12	12	11	11

^a^Effect size: The first letter is the size of *β*_*a*_, the second letter is the size of *β*_*b*_; *S* is small (0.14), *M* is medium (0.39), *L* is large (0.59) and *H* is halfway (0.26) between large and medium effect sizes

**Table 2:** Estimated numbers of required subjects for 2, 3, 4 and 5 observations with ICC = 0.2

Observations												
	2			3			4			5		
Effect size	Sobel	Product	Bootstrap	Sobel	Product	Bootstrap	Sobel	Product	Bootstrap	Sobel	Product	Bootstrap
SS	408	330	341	291	244	234	239	204	201	201	169	175
SH	301	282	283	231	226	230	201	185	190	177	163	160
SM	294	287	279	226	220	218	191	188	185	163	166	170
SL	282	267	278	223	213	211	188	182	182	166	163	164
HS	240	206	224	166	137	139	129	107	107	108	89	84
HH	120	97	104	87	72	79	71	59	58	61	53	60
HM	95	87	90	74	65	68	61	56	60	56	49	52
HL	88	85	85	72	65	70	61	57	54	51	49	48
MS	213	194	202	148	138	141	112	102	111	90	81	85
MH	81	68	73	59	49	53	47	39	37	39	32	30
MM	56	46	50	40	33	33	34	27	28	28	25	23
ML	47	38	37	32	31	28	29	27	23	25	24	24
LS	215	189	204	136	136	140	105	103	108	85	82	85
LH	66	60	65	45	39	40	36	32	34	30	26	28
LM	38	32	33	27	24	24	21	18	18	19	16	14
LL	25	22	22	19	17	15	16	14	12	14	12	13

**Table 3:** Estimated numbers of required subjects for 2, 3, 4 and 5 observations with ICC = 0.4

Observations												
	2			3			4			5		
Effect size	Sobel	Product	Bootstrap	Sobel	Product	Bootstrap	Sobel	Product	Bootstrap	Sobel	Product	Bootstrap
SS	479	394	395	363	300	334	313	269	276	282	251	253
SH	379	351	564	305	299	298	276	266	270	290	244	254
SM	376	350	351	307	301	302	269	251	253	282	238	232
SL	360	361	361	293	302	305	271	276	275	283	251	264
HS	258	215	219	181	153	159	149	120	134	137	106	111
HH	143	117	123	108	90	92	91	80	88	93	72	75
HM	116	109	111	96	88	88	85	79	81	88	72	78
HL	112	109	110	93	86	90	84	79	85	86	73	74
MS	217	210	212	152	132	141	116	105	118	100	83	85
MH	88	72	81	65	54	59	56	45	48	54	39	40
MM	64	54	55	49	45	47	42	37	38	42	35	33
ML	54	49	50	45	41	44	39	38	38	41	35	40
LS	209	198	204	143	138	140	105	96	108	85	81	87
LH	68	61	63	49	42	49	40	33	37	35	28	29
LM	41	34	36	32	25	26	25	22	21	24	19	19
LL	29	24	26	23	20	23	20	18	19	20	17	18

**Table 4:** Estimated numbers of required subjects for 2, 3, 4 and 5 observations with ICC = 0.6

Observations												
	2			3			4			5		
Effect size	Sobel	Product	Bootstrap	Sobel	Product	Bootstrap	Sobel	Product	Bootstrap	Sobel	Product	Bootstrap
SS	551	467	477	438	378	385	400	326	333	351	326	326
SH	451	426	453	376	369	370	363	349	350	332	326	334
SM	451	438	451	380	377	380	357	346	351	326	323	333
SL	454	444	445	385	376	380	344	326	340	326	313	324
HS	289	234	239	200	171	179	171	136	157	145	120	127
HH	156	132	130	127	111	122	111	101	108	104	93	96
HM	142	125	132	115	111	113	107	102	105	100	98	98
HL	133	131	127	115	111	112	102	97	97	100	97	99
MS	226	194	202	157	145	148	129	108	118	106	88	85
MH	99	80	91	76	62	73	63	52	60	57	48	52
MM	72	62	70	61	51	53	54	47	48	49	46	48
ML	63	59	61	53	52	52	49	49	49	47	46	46
LS	211	201	204	138	132	135	117	105	108	91	79	87
LH	72	62	65	52	45	49	43	36	38	37	31	32
LM	45	38	39	35	29	34	30	24	28	26	23	24
LL	33	31	32	28	24	25	25	24	25	23	21	23

**Table 5:** Estimated numbers of required subjects for 2, 3, 4 and 5 observations with ICC = 0.9

Observations												
	2			3			4			5		
Effect size	Sobel	Product	Bootstrap	Sobel	Product	Bootstrap	Sobel	Product	Bootstrap	Sobel	Product	Bootstrap
SS	638	576	581	544	495	523	499	465	454	476	426	455
SH	551	545	550	499	468	473	462	463	464	458	440	465
SM	551	550	550	507	485	490	476	479	478	444	447	445
SL	568	576	577	512	499	500	461	466	466	447	425	435
HS	308	246	251	236	189	204	193	163	168	175	148	162
HH	195	165	170	159	144	149	144	139	142	143	129	134
HM	183	171	170	143	148	149	139	133	132	134	128	129
HL	167	164	166	154	145	148	134	134	132	134	129	131
MS	232	208	212	171	145	153	136	113	111	115	95	102
MH	109	92	102	90	74	85	79	66	70	71	65	67
MM	86	76	82	73	66	65	65	64	64	63	60	62
ML	82	74	77	70	65	66	63	63	63	65	61	62
LS	213	207	216	144	133	137	111	108	109	91	84	84
LH	77	64	76	56	48	54	49	40	48	43	36	40
LM	50	42	46	41	36	34	35	31	32	32	27	30
LL	41	34	37	33	31	33	31	29	30	30	28	30

Among the three methods of testing the mediation effects, Sobel’s method required the largest sample size to achieve 80% power. Bootstrapping and the distribution of the product method performed similarly and were more powerful than Sobel’s method, as reflected by the relatively smaller sample sizes. For instance, when the mediation effect size was medium (i.e., SM) and the ICC was 0.2, with 4 repeated measures, Sobel’s method required 191 subjects to achieve 80% power, whereas the distribution of the product and bootstrap methods required 188 and 185 subjects, respectively, to achieve the same power.

For all three methods, the sample size required to achieve 80% power depended on the value of the ICC (i.e., within-subject correlation). A larger value of ICC typically required a larger sample size to achieve 80% power. For example, under the design with two repeated measures and using the distribution of the product method, to detect a small effect size of SS, a sample size of 299 was needed when ICC = 0.1, while a sample size of 420 was needed when ICC = 0.4.

Simulation results also illustrated the advantage of the longitudinal study design. Compared with the results reported by Fritz and MacKinnon [14] for the cross-sectional study, the required sample size under the longitudinal design was substantially smaller. When the ICC was low, such as 0.1, the required sample size under the longitudinal study design was a fraction of that under the cross-sectional design, and was approximately equal to the sample size of the cross-sectional study divided by the number of repeated measures. For example, under the longitudinal design with three repeated measures and using the distribution of the product method, the sample size under the longitudinal design was 215 to detect a small effect size of SS, which was approximately one-third of that required under the cross-sectional design (667). Even when the ICC was relatively high, we still observed dramatic sample size savings. For example, when ICC = 0.6 and using the bootstrap method, to detect the mediation effect size SM, the cross-sectional design required 422 subjects, while the longitudinal design with 4 repeated measures only required 351 subjects. This observation is in accordance to findings in literatures [19].

**Fig. 3:**
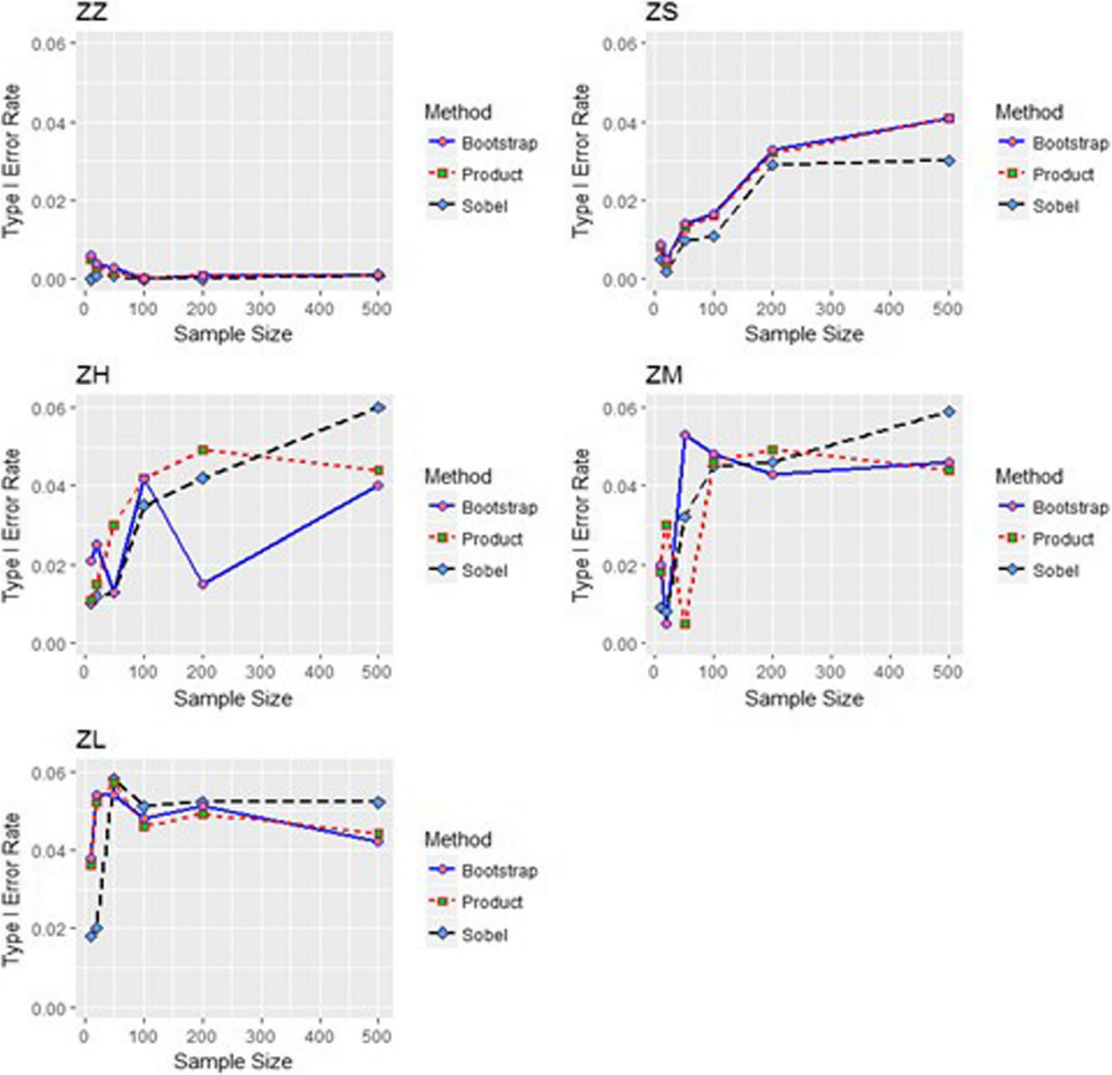
Type I error rates of Sobel’s (black line), distribution of the product (red line), and bootstrap (blue line) methods under various sample sizes with 3 repeated measures and ICC of 0.3

Figure 3 shows the type I error rates for the sample sizes corresponding to 5 examples of zero mediation effects when ICC = 0.3 for three repeated measures. A parameter combination of zero/zero (ZZ) had error rates around zero for all numbers of observations and sample sizes across the mediation tests. The distribution of the product method had the most precise rates; whereas Sobel’s method had less type I error probability and bootstrapping inflated the error rates in the case of a zero/0.59 (ZL) parameter, as with small sample sizes. However, the rates approached 0.05 when the number of sample sizes increased. Other scenarios taking various ICCs and repeated measures showed results similar to those in Fig. 3 and they are not shown in the paper.

## Discussion

Assuming a two-level mediation model and using Monte Carlo simulations, we determined the sample sizes required to achieve 80% power for longitudinal mediation studies under various practical settings. The simulation results provide guidance for researchers when choosing appropriate sample sizes in the design of longitudinal mediation studies. Our simulations also show that the distribution of the product and bootstrap methods are more powerful than Sobel’s method for testing the mediation effect. In addition, the required sample size is closely related to the ICC. A high ICC generally requires a larger sample size to detect a given effect size. The simulation results show that when the ICC is high, above 0.8 for instance, the required sample sizes in these scenarios are close to the values provided in Fritz et al. [14], suggesting that we should choose cross-sectional studies instead of longitudinal studies since the former is relatively easy to conduct but does not lose power. However, in real studies, especially in psychotherapy clinical trial studies, a meta-analysis of ICCs found that ICCs varied widely, ranging from 0 to 0.729, with an average around 0.08 [8]. Similar results have been found in clinical trial data [12, 20] and clinical practice data [24, 32, 37]. In studies in the field of education, small ICCs are also common [15], with 0.20 as a median value.

Another interesting finding for multilevel mediation is that the power of testing the mediation effect depends on not only the overall value of the mediation effects *β*_*a*_*β*_*b*_, but also the values of the individual regression coefficients *β*_*a*_ and *β*_*b*_. For instance, the sample size required to detect the effect size of LS is different from that required to detect the effect size SL. In other words, the sample size depends on the position of the effect sizes. Such a “positioning” effect for testing the mediation effect in multilevel mediation depends on the ICC. A high ICC leads to a stronger positioning effect. For example, in Table 5, when ICC = 0.9, detecting the effect size *SL* requires 568 subjects, while detecting the effect size *LS* only requires 213 subjects. The positioning effect does not appear in the single-level mediation model, which can be viewed as an extreme case of the multilevel model with ICC = 0. In the single-level mediation model, the required sample size (or power) only depends on the value of *β*_*a*_*β*_*b*_, but not the individual values of *β*_*a*_ and *β*_*b*_ [14]. For example, the number of subjects needed to detect the effect size *LS* was equal to that required to detect the effect size *SL*. The different behavior of multilevel mediation compared to single-level mediation is due to the within-cluster correlation in the multilevel model. Therefore, when conducting power calculations for longitudinal mediation studies, in addition to the mediation effect *β*_*a*_*β*_*b*_, it is equally important to report the effect size of *β*_*a*_ and *β*_*b*_.

Our simulation studies showed that the bootstrap and the distribution of the product methods have similar performance in testing the mediation effect. However, as the bootstrap is much more computer-intensive and time-consuming, we recommend using the distribution of the product method in practice. One limitation is that in the paper, coefficients *β*_*c*_, *β*_*a*_, *β*_*b*_ and *β*_*c*′_ in the model were treated as fixed-effects coefficients only. More flexible model by treating these as random-effects variables and two-level random-slopes model can also be considered. Another limitation is that in practice, effects size estimates are just estimates, not the true values, so uncertainty needs to be considered in the effect size estimates for sample size planning. Interested readers can consult the papers by [2, 10] for more information. There is a recent paper [38] discusses power and sample size for mediation model in longitudinal studies, however, in their model, the mediator was assumed to be time-invarying instead of time-variant in our research.

## Conclusion

Mediation analysis using longitudinal data allows researchers to investigate biological pathways and identifies their direct and indirect contribution to interested outcome variable. However, though this method is common in psychological and social research, sample size determination is still a challenging problem. This paper gives a way of using multilevel model for longitudinal data to provide the sample size under various sizes of the mediation effect, within-subject correlations and numbers of repeated measures via simulations by using three methods, Sobel, distribution of product and bootstrap. We found that the bootstrap and distribution of the product methods had comparable results and were more powerful than the Sobel’s method in terms of relatively smaller sample sizes. We recommend to use the distribution of product method due to its less computational load. For the mediation model of longitudinal data, the sample size depended on the ICC (i.e., the intra-subject correlation), number of repeated measurements, “position” of *β*_*a*_* and β*_*b*_. Sample size tables for commonly encountered scenarios in practice were also provided for researchers’ convenient use.

## Additional file

Additional file 1: Estimated numbers of required subjects with ICC = 0.3, 0.5, 0.7 and 0.8. (DOCX 27 kb)
