# Supplementary material for: Sample size determination for mediation analysis of longitudinal data
Source: BMC Med Res Methodol. 2018 Mar 27;18:32. doi: 10.1186/s12874-018-0473-2 (PMC5870539; doi:10.1186/s12874-018-0473-2)
Supplement: Supplementary file 1 — Estimated numbers of required subjects with ICC = 0.3, 0.5, 0.7 and 0.8. (DOCX 27 kb) [file 12874_2018_473_MOESM1_ESM.docx]

**Supplementary Tables for ICC equals to 0.3, 0.5, 0.7, 0.8**

**S 1**. Estimated numbers of required subjects for 2, 3, 4 and 5 observations with ICC = 0.3.

|  | | Observations | | | | | | | | | | | | | | | |
| --- | --- | --- | --- | --- | --- | --- | --- | --- | --- | --- | --- | --- | --- | --- | --- | --- | --- |
|  |  |  | 2 |  |  |  | 3 |  |  |  | 4 |  |  |  | 5 |  |  |
| Effect size |  | Sobel | Product | Bootstrap |  | Sobel | Product | Bootstrap |  | Sobel | Product | Bootstrap |  | Sobel | Product | Bootstrap |  |
| SS |  | 438 | 363 | 413 |  | 326 | 272 | 330 |  | 270 | 226 | 253 |  | 343 | 213 | 223 |  |
| SH |  | 344 | 326 | 323 |  | 274 | 257 | 262 |  | 230 | 226 | 228 |  | 209 | 207 | 210 |  |
| SM |  | 337 | 332 | 334 |  | 263 | 250 | 248 |  | 224 | 226 | 227 |  | 208 | 200 | 204 |  |
| SL |  | 313 | 326 | 330 |  | 261 | 263 | 260 |  | 232 | 219 | 225 |  | 204 | 201 | 201 |  |
| HS |  | 247 | 200 | 209 |  | 177 | 142 | 152 |  | 140 | 115 | 127 |  | 117 | 97 | 98 |  |
| HH |  | 126 | 107 | 115 |  | 97 | 84 | 89 |  | 81 | 68 | 70 |  | 72 | 64 | 67 |  |
| HM |  | 105 | 97 | 101 |  | 85 | 79 | 81 |  | 74 | 65 | 69 |  | 63 | 61 | 62 |  |
| HL |  | 100 | 98 | 99 |  | 79 | 77 | 78 |  | 70 | 68 | 70 |  | 61 | 61 | 61 |  |
| MS |  | 219 | 201 | 211 |  | 150 | 134 | 138 |  | 111 | 102 | 111 |  | 91 | 82 | 85 |  |
| MH |  | 84 | 72 | 80 |  | 62 | 52 | 56 |  | 51 | 41 | 40 |  | 42 | 35 | 36 |  |
| MM |  | 59 | 49 | 59 |  | 45 | 39 | 48 |  | 38 | 33 | 42 |  | 34 | 31 | 35 |  |
| ML |  | 51 | 44 | 54 |  | 39 | 38 | 45 |  | 33 | 31 | 32 |  | 31 | 30 | 27 |  |
| LS |  | 209 | 201 | 211 |  | 137 | 139 | 138 |  | 101 | 104 | 103 |  | 85 | 80 | 85 |  |
| LH |  | 71 | 59 | 58 |  | 48 | 40 | 43 |  | 38 | 31 | 34 |  | 38 | 25 | 37 |  |
| LM |  | 40 | 33 | 36 |  | 28 | 24 | 29 |  | 24 | 19 | 23 |  | 24 | 17 | 21 |  |
| LL |  | 27 | 24 | 26 |  | 21 | 18 | 23 |  | 18 | 16 | 20 |  | 17 | 15 | 17 |  |

**S 2**. Estimated numbers of required subjects for 2, 3, 4 and 5 observations with ICC = 0.5.

|  | | Observations | | | | | | | | | | | | | | | |
| --- | --- | --- | --- | --- | --- | --- | --- | --- | --- | --- | --- | --- | --- | --- | --- | --- | --- |
|  |  |  | 2 |  |  |  | 3 |  |  |  | 4 |  |  |  | 5 |  |  |
| Effect size |  | Sobel | Product | Bootstrap |  | Sobel | Product | Bootstrap |  | Sobel | Product | Bootstrap |  | Sobel | Product | Bootstrap |  |
| SS |  | 515 | 420 | 442 |  | 397 | 326 | 381 |  | 347 | 307 | 308 |  | 326 | 288 | 292 |  |
| SH |  | 435 | 404 | 421 |  | 363 | 338 | 345 |  | 319 | 288 | 293 |  | 290 | 282 | 292 |  |
| SM |  | 402 | 390 | 398 |  | 343 | 332 | 343 |  | 302 | 294 | 301 |  | 282 | 300 | 292 |  |
| SL |  | 400 | 390 | 392 |  | 335 | 331 | 334 |  | 310 | 301 | 303 |  | 283 | 274 | 282 |  |
| HS |  | 270 | 218 | 219 |  | 195 | 159 | 160 |  | 160 | 126 | 143 |  | 137 | 108 | 122 |  |
| HH |  | 155 | 130 | 136 |  | 119 | 100 | 109 |  | 93 | 90 | 91 |  | 93 | 82 | 84 |  |
| HM |  | 128 | 127 | 126 |  | 105 | 100 | 102 |  | 97 | 88 | 96 |  | 88 | 88 | 83 |  |
| HL |  | 129 | 128 | 129 |  | 103 | 97 | 99 |  | 122 | 88 | 97 |  | 86 | 86 | 83 |  |
| MS |  | 218 | 201 | 200 |  | 157 | 132 | 158 |  | 118 | 101 | 119 |  | 100 | 84 | 101 |  |
| MH |  | 95 | 79 | 79 |  | 70 | 61 | 70 |  | 49 | 49 | 49 |  | 54 | 45 | 49 |  |
| MM |  | 68 | 57 | 61 |  | 54 | 47 | 53 |  | 45 | 45 | 45 |  | 42 | 39 | 42 |  |
| ML |  | 56 | 53 | 55 |  | 49 | 46 | 47 |  | 42 | 42 | 42 |  | 41 | 41 | 41 |  |
| LS |  | 210 | 199 | 205 |  | 141 | 137 | 139 |  | 106 | 102 | 105 |  | 85 | 83 | 83 |  |
| LH |  | 72 | 61 | 67 |  | 49 | 42 | 45 |  | 40 | 34 | 35 |  | 35 | 28 | 31 |  |
| LM |  | 42 | 36 | 39 |  | 32 | 28 | 30 |  | 28 | 23 | 24 |  | 24 | 21 | 24 |  |
| LL |  | 31 | 28 | 29 |  | 25 | 23 | 24 |  | 23 | 22 | 23 |  | 20 | 19 | 20 |  |

**S 3.** Estimated numbers of required subjects for 2, 3, 4 and 5 observations with ICC = 0.7.

|  | | Observations | | | | | | | | | | | | | | |
| --- | --- | --- | --- | --- | --- | --- | --- | --- | --- | --- | --- | --- | --- | --- | --- | --- |
|  |  |  | 2 |  |  |  | 3 |  |  |  | 4 |  |  |  | 5 |  |
| Effect size |  | Sobel | Product | Bootstrap |  | Sobel | Product | Bootstrap |  | Sobel | Product | Bootstrap |  | Sobel | Product | Bootstrap |
| SS |  | 575 | 498 | 503 |  | 477 | 400 | 402 |  | 426 | 379 | 389 |  | 396 | 363 | 365 |
| SH |  | 450 | 420 | 423 |  | 441 | 426 | 422 |  | 394 | 308 | 390 |  | 376 | 358 | 365 |
| SM |  | 488 | 477 | 481 |  | 426 | 424 | 425 |  | 376 | 376 | 376 |  | 376 | 366 | 370 |
| SL |  | 469 | 455 | 459 |  | 418 | 416 | 418 |  | 376 | 365 | 370 |  | 363 | 350 | 355 |
| HS |  | 291 | 234 | 252 |  | 211 | 174 | 185 |  | 184 | 141 | 144 |  | 159 | 127 | 129 |
| HH |  | 171 | 146 | 152 |  | 138 | 120 | 122 |  | 127 | 114 | 121 |  | 120 | 116 | 112 |
| HM |  | 155 | 144 | 150 |  | 130 | 123 | 126 |  | 116 | 116 | 116 |  | 111 | 106 | 108 |
| HL |  | 146 | 143 | 144 |  | 126 | 121 | 123 |  | 116 | 116 | 116 |  | 105 | 107 | 105 |
| MS |  | 231 | 205 | 216 |  | 163 | 140 | 151 |  | 129 | 108 | 111 |  | 107 | 92 | 105 |
| MH |  | 102 | 84 | 93 |  | 80 | 65 | 71 |  | 68 | 56 | 59 |  | 61 | 51 | 58 |
| MM |  | 78 | 70 | 72 |  | 64 | 54 | 60 |  | 57 | 55 | 57 |  | 54 | 49 | 51 |
| ML |  | 69 | 70 | 70 |  | 61 | 57 | 60 |  | 54 | 51 | 51 |  | 52 | 51 | 51 |
| LS |  | 210 | 210 | 210 |  | 141 | 129 | 138 |  | 111 | 103 | 104 |  | 87 | 82 | 83 |
| LH |  | 74 | 64 | 71 |  | 55 | 45 | 46 |  | 46 | 37 | 44 |  | 40 | 32 | 33 |
| LM |  | 47 | 40 | 41 |  | 37 | 31 | 35 |  | 32 | 26 | 23 |  | 28 | 24 | 26 |
| LL |  | 36 | 33 | 34 |  | 29 | 25 | 27 |  | 26 | 25 | 25 |  | 25 | 24 | 24 |

**S 4**. Estimated numbers of required subjects for 2, 3, 4 and 5 observations with ICC = 0.8.

|  | | Observations | | | | | | | | | | | | | | |
| --- | --- | --- | --- | --- | --- | --- | --- | --- | --- | --- | --- | --- | --- | --- | --- | --- |
|  |  |  | 2 |  |  |  | 3 |  |  |  | 4 |  |  |  | 5 |  |
| Effect size |  | Sobel | Product | Bootstrap |  | Sobel | Product | Bootstrap |  | Sobel | Product | Bootstrap |  | Sobel | Product | Bootstrap |
| SS |  | 620 | 521 | 528 |  | 499 | 457 | 458 |  | 461 | 426 | 426 |  | 438 | 401 | 398 |
| SH |  | 557 | 521 | 553 |  | 469 | 450 | 460 |  | 426 | 426 | 426 |  | 408 | 401 | 402 |
| SM |  | 550 | 513 | 543 |  | 469 | 460 | 459 |  | 426 | 401 | 411 |  | 394 | 394 | 394 |
| SL |  | 544 | 519 | 535 |  | 482 | 463 | 471 |  | 426 | 419 | 421 |  | 413 | 401 | 403 |
| HS |  | 303 | 240 | 239 |  | 225 | 181 | 199 |  | 186 | 153 | 164 |  | 166 | 135 | 145 |
| HH |  | 185 | 162 | 170 |  | 150 | 139 | 145 |  | 134 | 134 | 134 |  | 134 | 119 | 125 |
| HM |  | 161 | 158 | 160 |  | 139 | 136 | 137 |  | 128 | 120 | 122 |  | 125 | 116 | 118 |
| HL |  | 159 | 148 | 150 |  | 134 | 134 | 134 |  | 133 | 126 | 131 |  | 118 | 113 | 114 |
| MS |  | 230 | 206 | 202 |  | 163 | 135 | 141 |  | 132 | 110 | 118 |  | 112 | 92 | 95 |
| MH |  | 109 | 87 | 91 |  | 86 | 70 | 77 |  | 70 | 62 | 67 |  | 66 | 55 | 60 |
| MM |  | 84 | 75 | 80 |  | 67 | 61 | 63 |  | 63 | 56 | 61 |  | 61 | 56 | 57 |
| ML |  | 74 | 66 | 67 |  | 63 | 61 | 62 |  | 58 | 58 | 58 |  | 56 | 51 | 54 |
| LS |  | 207 | 203 | 204 |  | 145 | 132 | 140 |  | 110 | 102 | 108 |  | 92 | 80 | 87 |
| LH |  | 76 | 64 | 65 |  | 56 | 48 | 52 |  | 47 | 40 | 41 |  | 41 | 34 | 37 |
| LM |  | 48 | 40 | 43 |  | 38 | 32 | 36 |  | 34 | 28 | 29 |  | 30 | 27 | 28 |
| LL |  | 37 | 34 | 36 |  | 32 | 27 | 30 |  | 29 | 26 | 27 |  | 27 | 26 | 26 |
